# Is precision medicine the solution to improve organ preservation in laryngeal/hypopharyngeal cancer? A position paper by the Preserve Research Group

**DOI:** 10.3389/fonc.2024.1433333

**Published:** 2024-08-06

**Authors:** Davide Mattavelli, Gunnar Wichmann, Davide Smussi, Alberto Paderno, Maria Serrahima Plana, Ricard Nin Mesia, Micaela Compagnoni, Alessandro Medda, Susanna Chiocca, Stefano Calza, Yinxiu Zhan, Carla Rognoni, Rosanna Tarricone, Erika Stucchi, Luigi Lorini, Cristina Gurizzan, Ksenia Khelik, Eivind Hovig, Andreas Dietz, Cesare Piazza, Paolo Bossi

**Affiliations:** ^1^ Unit of Otorhinolaryngology – Head and Neck Surgery, ASST Spedali Civili di Brescia, Brescia, Italy; ^2^ Department of Medical and Surgical Specialties, Radiological Sciences, and Public Health, University of Brescia, Brescia, Italy; ^3^ Clinic of Otolaryngology, Head and Neck Surgery, Department of Head Medicine and Oral Health, University Hospital Leipzig, Leipzig, Germany; ^4^ Unit of Medical Oncology, ASST Spedali Civili di Brescia, Brescia, Italy; ^5^ Department of Biomedical Sciences, Humanitas University (Milan), IRCCS Humanitas Research Hospital, Rozzano, Italy; ^6^ Medical Oncology Department, Institut Català d’Oncologia (ICO-Hospitalet), IDIBELL, Hospitalet de Llobregat, Barcelona, Spain; ^7^ Department of Experimental Oncology, IEO, European Institute of Oncology IRCCS, Milan, Italy; ^8^ Unit of Biostatistics and Bioinformatics, Department of Molecular and Translational Medicine, University of Brescia, Brescia, Italy; ^9^ Centre for Research on Health and Social Care Management (CERGAS), SDA Bocconi School of Management, Bocconi University, Milan, Italy; ^10^ Department of Social and Political Sciences, Bocconi University, Milan, Italy; ^11^ Medical Oncology and Hematology Unit, IRCCS Humanitas Research Hospital, Milan, Italy; ^12^ Department of Biomedical Sciences, Humanitas University, Milan, Italy; ^13^ Centre for Bioinformatics, Department of Informatics, University of Oslo, Oslo, Norway; ^14^ Department of Tumor Biology, Institute for Cancer Research, Oslo University Hospital, Oslo, Norway

**Keywords:** squamous cell carcinoma, larynx, hypopharynx, head and neck, chemotherapy, radiotherapy, organ preservation

## Abstract

In locally advanced (LA) laryngeal/hypopharyngeal squamous cell carcinoma (LHSCC), larynx preservation (LP) strategies aim at the cure of the disease while preserving a functional larynx, thus avoiding total laryngectomy and the associated impact on the quality of life. In the last decades, apart from transoral and open-neck organ preservation approaches, several non-surgical regimens have been investigated: radiotherapy alone, alternate, concurrent or sequential chemoradiation, and bioradiotherapy. Despite major progress, the identification of reliable and effective predictors for treatment response remains a clinical challenge. This review examines the current state of LP in LA-LHSCC and the need for predictive factors, highlighting the importance of the PRESERVE trial in addressing this gap. The PRESERVE trial represents a pivotal initiative aimed at finding the optimal therapy for laryngeal preservation specific to each patient through a retrospective analysis of data from previous LP trials and prospectively validating findings. The goal of the PRESERVE trial is to develop a comprehensive predictive classifier that integrates clinical, molecular, and multi-omics data, thereby enhancing the precision and efficacy of patient selection for LP protocols.

## 1 Introduction

Laryngeal and hypopharyngeal squamous cell carcinoma (LHSCC) is a rare form of cancer, accounting for only 0.8% of all new cancer cases (1). Nevertheless, it has significant social importance due to the vital role the larynx plays in the production of voice, swallowing, social functions, and overall quality of life (QoL).

Whereas early-stage LHSCC with T1-T2 tumors without regional metastasis (N0) and selected T3 cancers are candidates for either organ-preserving surgery or radiotherapy (RT) alone, these treatments may be insufficient in most patients suffering from loco-regional advanced (LA) LHSCC (2). Historically, the standard treatment for these cancers comprised total laryngectomy (TL) and neck dissection (ND) followed by adjuvant RT, often combined with cisplatin-based chemotherapy (CRT) according to pathological risk factors (3, 4). The loss of laryngeal functions due to TL has disruptive effects on the psychological, social, and intimate environment of the patient. Despite effective options for voice rehabilitation have been developed, TL may still have a detrimental effect on the QoL of patients and their relatives and cause depression and apathy (5–7).

In the Nineties, laryngeal preservation (LP) strategies for LA-LHSCC having TL as the only surgical option were investigated as an alternative. Briefly, non-surgical treatment protocols, including RT alone or in combination with chemotherapy, were tested to preserve a functioning larynx while granting non-inferior survival outcomes. The final goal of LP trials was to provide an alternative to TL for the highest possible percentage of patients with LA-LHSCC.

Three main protocols for LP have been developed so far: induction chemotherapy (IC) followed by RT, concurrent chemoradiotherapy (CCRT), and alternating chemoradiotherapy, whose use has progressively declined (4, 8).

Is there no chance to *a priori* discriminate between patients who are going to most benefit from IC and those who are not in order to improve LP outcomes, avoid useless treatments and reduce long-term toxicities? To date, patient selection for larynx preservation treatment rather than surgical non conservative treatment is carried out based on limited clinical and radiological characteristics. No predictive marker of response to induction chemotherapy, and therefore of success for the organ-preservation strategy, is currently known, and only the response to the first cycle IC can be used as a predictor (3, 4, 8, 9). Hence, there is an unmet need to improve patient selection by defining biomarkers predicting either response or resistance before starting the regimen, as well as defining potential response to new treatment strategies, as otherwise, their only remaining option is ablative surgery.

It is in this context that the PRESERVE trial emerges: a clinical study funded by Europe through the ERA PerMed project ERAPERMED2020–283 with the aim to evaluate the efficacy of a tailored treatment as laryngeal-hypopharyngeal preservation strategy and to find the prognostic and predictive value of biomarkers identified throughout the translational analysis to be utilized for personalized treatment of LA-LHSCC patients.

The purpose of the article is to provide an overview of the current scenario and to introduce the PRESERVE trial.

## 2 Treatment approaches

With the aim to prevent the functional loss related to TL without compromising survival or disease control, non-surgical LP approaches to treat LA-LHSCC patients have been extensively studied.

The first approach evaluated for LHSCC was IC with three cycles of cisplatin and 5-fluorouracil (PF) followed by RT in responders or by a TL followed by adjuvant RT in non-responders.

Two large, randomized phase III trials compared this strategy versus upfront TL with postoperative RT (9, 10). The larynx was preserved in 64% of patients with laryngeal tumors (9) and 42% of patients with hypopharyngeal tumors at 2 years (10), with no difference in survival outcomes. These two studies established a new standard of care for patients with both laryngeal and hypopharyngeal squamous cell carcinoma (SCC) candidates to TL, demonstrating that the larynx could be preserved in up to two-thirds of patients.

Overall, IC has the rationale of achieving tumor shrinkage, allowing better RT planning and reducing the risk for distant metastases. Moreover, in the last decade, it has been widely studied as a dynamic predictive biomarker to assess the likelihood of success with non-surgical LP strategies. In fact, multiple studies have demonstrated that a significant response after two or even one cycle(s) of IC (defined as a radiological response, i.e., a 50% or greater decrease in the sum of the product of the diameters of each measurable lesion) or relevant clinical improvement (i.e., recovery from vocal fold paralysis) closely correlates with favorable outcome after full-dose definitive RT (11, 12). This has led to the use of IC as a mean to select patients for non-surgical management, with those demonstrating a response to IC (complete [CR] or partial [PR] response) being treated with definitive RT and the others (stable [SD] or progressive [PD] disease) undergoing surgical resection followed by postoperative RT.

CCRT involves administering chemotherapy and RT concurrently, with the goal of increasing the effectiveness of both treatments (at least additively). To date, CCRT with cisplatin is recommended for advanced laryngeal cancer based on the results of the Intergroup trial RTOG 91–11 (13). This LP trial found that CCRT improved disease control rates until 5 years compared to IC followed by RT or RT alone.

LP was the primary endpoint, while laryngectomy-free survival (LFS) and overall survival (OS) were secondary endpoints. In the first report, the 2- and 5-year rates for LFS did not show significant differences between the IC and the CCRT arms (59% and 43% versus 66% and 45%, respectively). The rate of LP was significantly higher in survivors of the CCRT arm (84%) when compared with the IC arm (72%, *p*=0.005) or with the RT alone arm (67%, *p*<0.001). However, severe toxicity was more frequent with the CCRT schedule, and no difference in OS was observed. The 10.8-year follow-up update confirmed no significant difference in terms of LFS between the two arms containing chemotherapy and the superiority in LP rate in survivors of the CCRT arm. OS did not differ significantly between treatment arms, although there was a trend for a higher survival in the IC arm, probably secondary to late effects from CCRT that led to a significantly increased number of non-cancer-related deaths (14).

**Figure 1:**
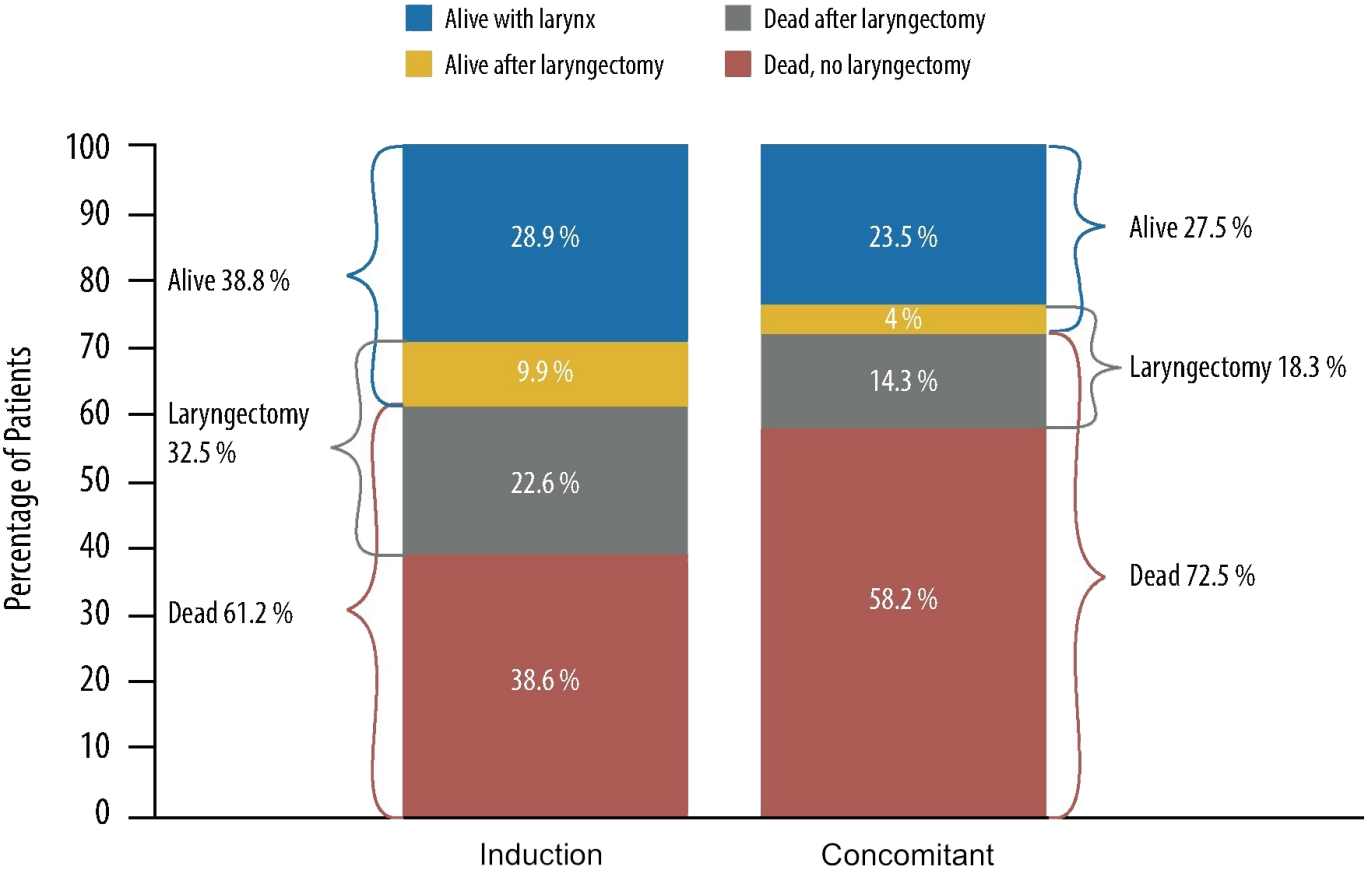
10-year outcome data of patients treated with induction cisplatin plus fluorouracil followed by radiotherapy and concurrent cisplatin (radiotherapy in RTOG [Radiation Therapy Oncology Group] 91–11 phase III randomized trial). Representation of RTOG 91–11 data from the re-analysis by Licitra et al. (15).

Finding the best protocol balancing survival and LFS with acceptable late toxicity and good functional outcome, and how to improve the frequency of LP without compromising OS is still a matter of debate. Giving more attention to functional aspects, the data of the RTOG 91–11 trial may suggest that IC is the best option for LP. Based on a recently published re-analysis of the RTOG 91–11, Licitra et al. stated that the trial’s results do not show any clinical superiority of CCRT over IC+RT regarding LP, suggesting that sequential treatment by applying IC+RT is more effective and achieves a substantial increase in the proportion of long-term survivors with and without the larynx (**Figure 1**) (15). As the chemotherapy scheme employed in the RTOG 91–11 is not the state-of-the-art induction treatment as of today, we may expect further benefits with the use of IC based on the taxane + PF (TPF) scheme.

Consistently, the two-arm randomized trial GORTEC 2000–01 showed that the addition of docetaxel to PF (TPF) achieved a statistically significant superior 3-year LP rate compared with the PF regimen (70.3% versus 57.5%; *p*=0.03). The TPF regimen obtained higher response rates, and both regimens were comparable in terms of late toxicity rates. No differences in terms of survival were found (16). The long-term evaluation confirmed the initial results (17). On the other hand, the addition of cetuximab in the IC regimen showed no superiority to IC with TPF/TP (3).

With regards to the best sequential treatment after IC in responders, whether the addition of systemic treatment to RT is advantageous is still to be determined. After induction of TPF, it can be difficult to administer high-dose cisplatin due to cumulative toxicity, and it raises the same concerns on long-term toxicities due to concurrent chemoradiation already discussed.

TREMPLIN, phase II, randomized trial compared cisplatin versus cetuximab concomitant with RT in patients responding to TPF and proved no differences in any efficacy endpoints, including LP and OS. However, the bioradiotherapy (BRT) arm showed a higher rate of local failure than with chemotherapy. Except for grade 1 renal toxicity, late toxicity did not differ significantly between both arms, although compliance was significantly superior in the BRT arm (18). Subsequently, the results of the TTCC 2007–02 trial performed by the Spanish Head and Neck Cancer Group showed similar results with BRT (19).

Based on current evidence, LA-LHSCC IC with TPF + RT or platinum-based CCRT are the most accepted treatment options when aiming at functional LP. Two main concerns are still unanswered. The first one is how to early identify patients who will not respond to IC (up to one-third according to previous studies) or CCRT. Hence, there is an unmet need to improve patient selection by defining biomarkers predicting either response or resistance before starting the regimen, as well as defining potential response to new treatment strategies, as otherwise, their only remaining option is ablative surgery. The other issue is whether IC with a TPF regimen can improve the outcomes obtained with CCRT. We are awaiting the results of the GORTEC 2014–03-SALTORL randomized LP trial that compares both strategies (NCT03340896) (20, 21).

**Table 1** summarizes the ongoing trials investigating IC for LP in LHSCC.

**Table 1:** Ongoing studies on induction chemotherapy in larynx-hypopharynx cancer.

NCT (acronym)	Disease site	IC drugs	Study phase	Primary endpoint	Sample	Status	Study completion
NCT01633541	Larynx	TP+AT101 (anti-Bcl)	II	3-month LEDFS	42	Completed	12/2021
NCT04539600	Hypopharynx	T + camrelizumab (anti-PD-1)	II	1-year PFS	23	Completed	12/2022
NCT04156698	Hypopharynx	TPF + camrelizumab	II	ORR	51	Completed	12/2022
NCT03894891	Larynx, hypopharynx	TP + Nivo (RT + Nivo)	II	2-year LEDFS	70	Completed	11/2024
NCT04943445 (SMART KEY)	Larynx	PF + Pembro	II	2-year LEDFS	42	Recruiting	12/2024
NCT05551767 (HN01)	Oropharynx p16-, larynx, hypopharynx: CPS ≥1	TP + Nivo	II	ORR + 3-year PFS	120	Recruiting	12/2024
NCT04926753 (DETECTOR1)	Larynx, hypopharynx	PF + toripalimab	I	ORR	20	Recruiting	06/2024
NCT04030455	Larynx, hypopharynx	TP + Pembro (four cycles)	II	CBR (after two cycles) + pCR	25	Recruiting	01/2025
NCT04995120 (INSIGHT)	Larynx, hypopharynx	TP + toripalimab	II	3-month LEDFS	42	Recruiting	12/2025
NCT06137378 (ELOS)	Larynx, hypopharynx CPS ≥1	TP ± 17 cycles pembrolizumab	IIB	LFS	140	Open, recruiting	12/2028

TP, taxane + platinum; PF, platinum + 5-fluorouracil; TPF, taxane + platinum + 5-fluorouracil; nivo, nivolumab; pembro, pembrolizumab; pCR, pathologic complete remission; ORR, overall response rate; LEDFS, laryngo–esophageal dysfunction-free survival; PFS, progression-free survival; LFS, laryngectomy-free survival; CBR, clinical benefit rate.

**Figure 2:**
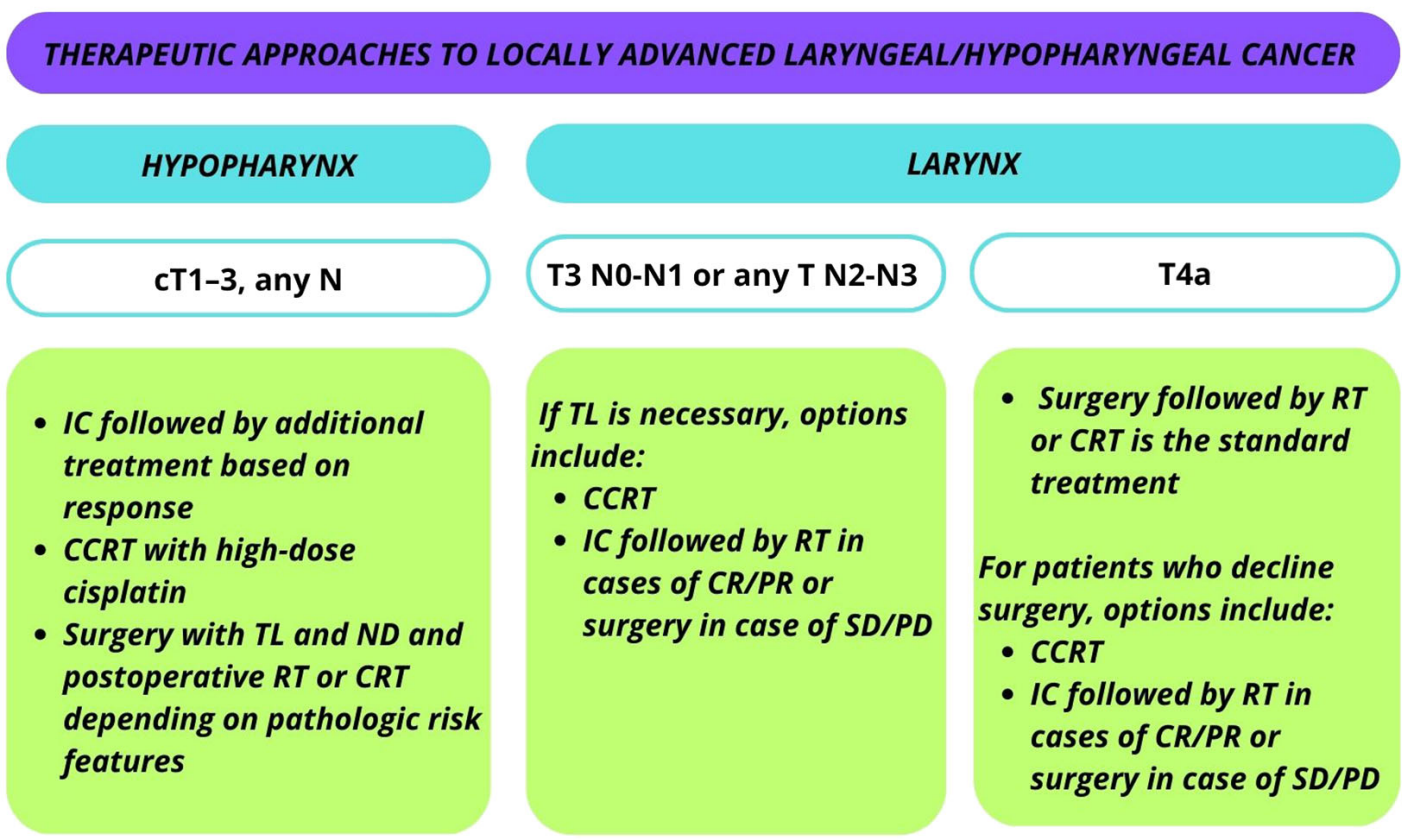
Current recommendations from the European Society for Medical Oncology (ESMO) and the Americas Society of Clinical Oncology (ASCO) for the application of LP strategies in the management of locally advanced (LA) laryngeal/hypopharyngeal cancer.

Current recommendations from both the European Society for Medical Oncology (ESMO) and the Americas Society of Clinical Oncology (ASCO) advocate the application of LP strategies in the management of LA, laryngeal/hypopharyngeal cancer are summarized in **Figure 2**.

For laryngeal T3 N0-N1 or T1–3 N2-N3 cases, concurrent chemoradiotherapy (CCRT) or organ preserving surgery is the standard treatment. In cases where TL is necessary, options include CCRT or IC followed by RT in cases of complete or partial response. Surgery comprising TL and ND is considered in the event of SD or PD after induction.

For T4a laryngeal cancer, the standard treatment involves TL and ND followed by RT or CCRT. Patients who decline surgery may opt for CCRT or IC with subsequent management based on response. Participation in organ preservation clinical trials is also an option.

In the case of cT1–3, any N category hypopharyngeal cancer, patients with surgical options of total laryngopharyngectomy have three main approaches: 1) IC followed by additional treatment based on response; 2) surgery with TL and ND and postoperative RT or CRT depending on pathologic risk features; or 3) CCRT with high-dose cisplatin (21).

## 3 Molecular alterations leading to LHSCC

LHSCC can result from a combination of genetic alterations and environmental influences: variants in genes involved in alcohol and tobacco processing and nucleotide excision repair pathways may increase cancer risk. Additionally, mutations in genes regulating cellular processes like proliferation, survival, and differentiation contribute to tumoral transformation (22, 23).

The tumor suppressor protein p53, often dysfunctional in LHSCC (24), correlates with advanced disease, especially locoregional metastasis (25), and poor prognosis when overexpressed along with MDM2 (26, 27). Similarly, overexpression of the anti-apoptotic protein BCL-2 is associated with aggressive tumor behavior resistant to therapies (28). Dysregulation of cell cycle proteins like P27 and cyclin D1 is common in LHSCC, with low P27 levels indicating aggressive disease and high cyclin D1 levels correlating with poor survival rates (29, 30).

Epidermal growth factor receptor (EGFR) overexpression destabilizes the cellular microenvironment and correlates with LHSCC aggressiveness (31). Targeting EGFR with cetuximab or tyrosine kinase inhibitors has potential, but interaction with anaplastic lymphoma kinase (ALK) may compromise therapy response (32). Dysregulation of transforming growth factor-β (TGF-β) signaling promotes cell proliferation and epithelial-to-mesenchymal transition (EMT), contributing to metastasis formation in LHSCC (33). Downregulation of epithelial markers and up-regulation of mesenchymal markers indicate advanced and metastatic disease (33, 34).

It was recently demonstrated that polymorphisms in human leukocyte antigen (HLA), especially in HLA-B antigens and homozygosity in HLA-Cw, DRB4, and haplotype combinations, are risk factors for HNSCC and affect progression-free survival (PFS) of HNSCC (35). Using the natural logarithm of the hazard ratio (HR) for PFS of eight HLA traits to calculate an HLA-score for the individual patient allowed for predicting PFS in the training cohort and in an independent validation cohort solely including LHSCC from the RCT DeLOS-II (36). Consequently, the HR linked to the presence (or absence) of detrimental or protective HLA antigens or haplotypes seems to be of critical relevance also for LP protocols.

## 4 Overview of current research based on immunotherapy in LHSCC

Based on the results from CheckMate-141 and the KEYNOTE-012, -040, and -048 RCTs, immune checkpoint inhibitors (ICI) nivolumab and pembrolizumab have already received approval from the “Food and Drug Administration” and “European Medicines Agency” as monotherapy for the treatment of recurrent or metastatic (R/M) HNSCC in adults progressing on or after platinum-containing chemotherapy (37–42). In the KEYNOTE-048 trial, the differential benefit has been shown in relationship with the expression level of PD-L1 according to combined positive score (CPS) and the treatment with pembrolizumab was employed both in monotherapy as well as in combination with chemotherapy, with favorable results (42).

The use of immunotherapy in trials dedicated to LHSCC is limited, with only a few recently reported experiences in small phase II trials of induction therapy comprising ICI. A combination of platinum, docetaxel and pembrolizumab has been administered to 24 patients as a single modality treatment for laryngeal preservation, with a pathological complete response (pCR) rate as a co-primary endpoint after four cycles (43). 77.3% of the patients obtained a pCR, of whom 35% developed a recurrence, thus highlighting at the same time the high sensitivity to this combination and the need to precisely define – clinically and immunologically – the subgroup of patients who could benefit from such a strategy.

Other small phase II trials have evaluated the activity of chemo-immunotherapy in the induction setting of locally advanced LHSCC before locoregional treatments. Overall response rate was achieved in 85–92% of the cases, with a 1-year LP rate between 79 and 92% (44, 45). These results need to be confirmed in larger trials, with longer follow up and the appreciation of the added long-term benefit to apply such a strategy to non-selected locally advanced LHSCC patients.

It should also be remembered that response to ICI requires functional cooperation between immune cells and the binding of either cytotoxic T cells or natural killer (NK) cells to tumor cells. While the first requires the presentation of tumor-associated antigens (TAA)-derived peptides in major histocompatibility (MHC) class I- and II-proteins (HLA-A, B or C and DP, DQ or DR, respectively) for activating T cells and their proliferation, the second requires the presence of TAA-derived peptides in MHC to allow for deleting tumor cells. This cooperation between different actors of the immune system is crucial in determining the prognosis and the possibility of observing responses to ICI. Moreover, the tumor microenvironment (TME) has a direct role in promoting or hampering the immune response. A hypoxic TME, with anaerobic metabolism, a high number of regulatory tumor-infiltrating lymphocytes and an increased angiogenesis are linked to a poor immune response (46).

## 5 Clinical predictors of response to LP strategies

Currently, the decision to candidate or not an LA-LHSCC patient for an LP protocol is difficult. The response to treatment is not obvious and depends on the complex interplay of clinical and molecular features of the tumor and neck nodes themselves. Additionally, patient performance status, the patient’s needs and desires, the experience and recommendation of the treating physicians, and the philosophy of the multidisciplinary tumor board are relevant to the outcome. Likewise, current views on long-term survival with functional organ preservation and still high failure rates are highlighting the need for better selection of patients, as emerged in the recently published data of the LP trials TREMPLIN (18) and DeLOS-II (3, 4) and their subgroup analyses (47).

The lack of reliable predictors of efficacy for LP strategies represents a relevant unmet clinical need. As previously mentioned, response to IC is currently the best dynamic option for patients’ selection; however, this approach is flawed by different limitations: a) the non-tailored administration of IC to all patients inevitably exposes the non-responders to unnecessary toxicities and delays the start of the correct treatment plan; b) IC could be contraindicated for patients’ comorbidities. On the other hand, CCRT can expose non-responders to an inappropriate treatment strategy for a substantially prolonged time with a higher risk of complications when salvage surgery is required (48). In this context, molecular and genomic markers may provide tumor fingerprints of responsiveness to (CC)RT, thus anticipating the probability of successful LP strategies. In fact, the application of recent advances in molecular tumor characterization, somatic mutations and methylation patterns in its DNA and transcriptomic gene-expression signatures (GES), as well as feature selection from radiologic investigations of tumor and metastasis (i.e., radiomic signatures or RS), may offer benefits to the decision making. A pre-requisite for this is the knowledge about RS, GES and gene variants or other biomarkers particularly linked to response or non-response of LHSCC to specific treatments that could be identified *in vitro* and/or *in vivo*, at best, using patient data from clinical trials supplemented with molecular characterization.

Several baseline patient- and tumor-dependent characteristics have been correlated in retrospective series to response to induction chemotherapy and LP. Among patients’ characteristics, comorbidity (assessed according to the Charlson comorbidity index, CCI), frailty, patient performance status (according to the ECOG or Karnofsky index), and lifestyle-related risk factors, including tobacco smoking and alcohol abuse are the most informative. In univariate analyses, CCI >0, ECOG >1, >30 pack-years, and >30 g/day alcohol consumption have been associated with reduced response rates and lower LFS and OS (5, 47). Similarly, blood parameters (i.e., platelet/lymphocyte ratio or neutrophils/lymphocyte ratio) (49, 50) and poor nutritional status at baseline (51) proved their predictive role in retrospective cohorts, but a prospective validation is still lacking.

According to disease characteristics, hypopharyngeal subsite, higher T-N categories and volume, high tumor metabolic rate and molecular features of the tumor have been associated with worse prognosis (13, 14, 25, 52–56).

Lefebvre and Ang reported key issues and recommendations for LP clinical trial design and provided ground for excluding particular subgroups. According to these key findings agreed in a consensus panel, they defined the T4 stage as a negative predictor of LP and, therefore as a possible exclusion criterion (57). However, even in these cases, using early response evaluation after the first cycle of IC and applying the “LFS score” to assess the probability of long-lasting good outcomes might raise the number of patients with preserved larynx (47). The LFS score consists of four independent parameters to predict LFS in early responders (after the first cycle). These are: 1) the number of clinically positive nodes (cut-off, 2); 2) the residual primary tumor volume (cut-off, 20%); 3) the residual total tumor volume (cut-off, 5.6 mL); 4) the ratio of residual SUVmax and SUVmean (cut-off, 1.51). In the formula, each parameter is weighted by its hazard ratio (12, 6, 5 and 4, respectively); LFS score ≤16 predicts increased LFS, OS, and tumor-specific survival (*p*<0.05) (47).

## 6 The PRESERVE trial

In our study, a few of European groups within the collaborative project PRESERVE in the ERA-Net framework. The project is composed of a retrospective and a prospective phase. Overall, we aim at identifying responders and non-responders and patients who could benefit in terms of laryngo-esophageal functional preservation upon treatment within LP protocols. Based on this, we designed a multicenter international trial to prove the feasibility of a stratified IC treatment approach for organ preservation in LHSCC patients (prospective trial) tailored according to clinical, GES, and RS information (obtained in the retrospective analysis).

**Figure 3:**
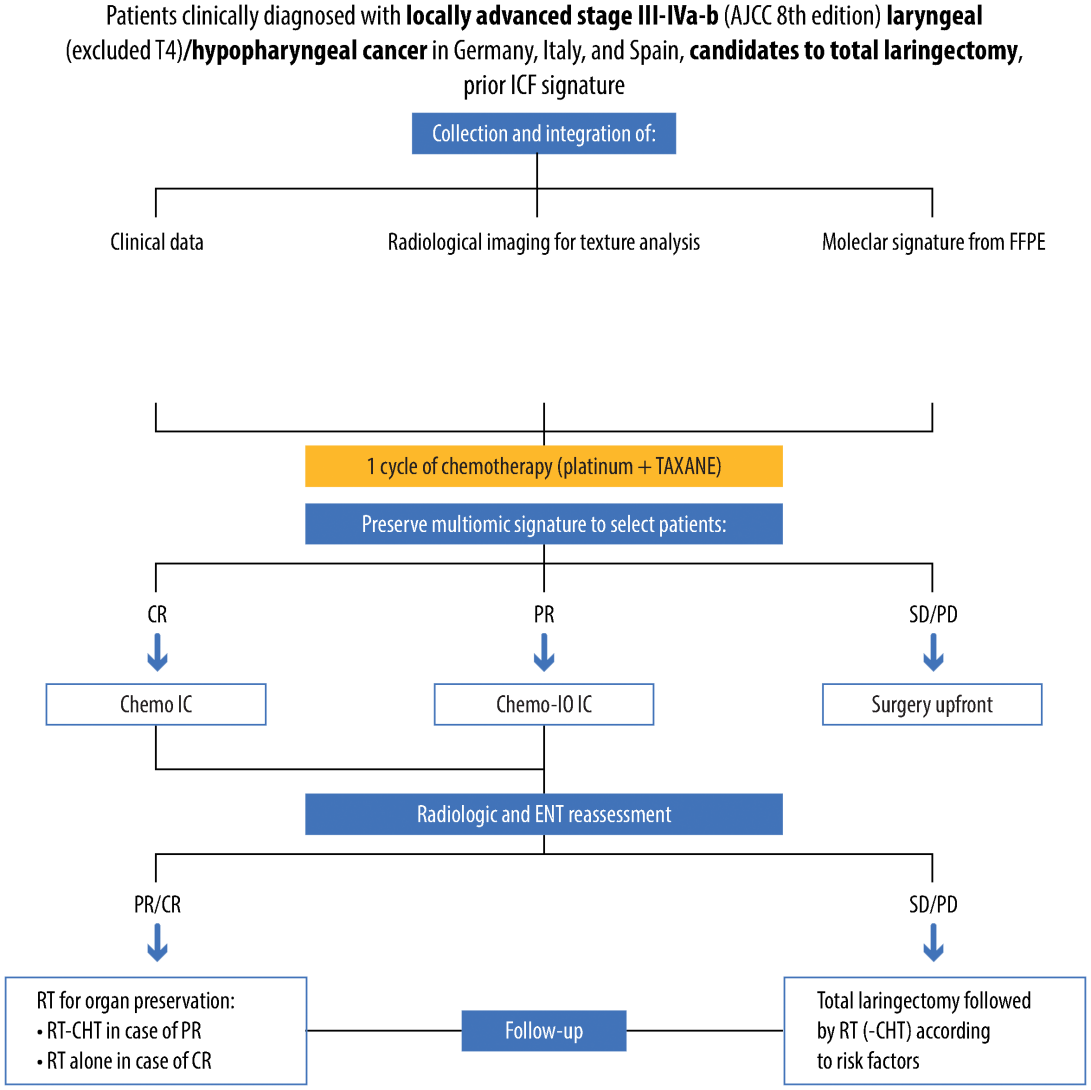
Detailed design of the clinical PRESERVE trial, reporting selected population, methods of analysis, performed treatments and therapies, results and follow-up of patients. Specifically, patients affected by stage III-IV, non-metastatic, laryngeal/hypopharyngeal cancer candidates to total laryngectomy will be enrolled. For all patients, a multiomic assessment including clinical factors, radiomic and genomic analysis will be performed, providing a prediction of response to induction chemotherapy. Patients predicted with partial response (PR) will receive chemo-immunotherapy induction treatment. After three cycles, patients will undergo endoscopical and radiological restaging and receive curative treatment according to a tailored strategy, and subsequent follow-up.

The PRESERVE clinical trial is a phase II, open-label, non-randomized, multicenter trial aiming to tackle this unmet clinical need by creating a multi-omics signature that combines clinical characteristics, radiomics, and genomics data (**Figure 3**).

### 6.1 Study design

This multi-omics signature will be built starting from the retrospective analysis of about 250 cases of locally advanced LHSCC initially treated with IC in the last 15 years. After obtaining informed consent from the patient, slides from the formalin-fixed paraffin-embedded (FFPE) specimen or snap-frozen tissue, if available, will undergo genomic analysis to extract total ribonucleic acid (RNA). After measuring RNA quality and concentration, we will generate a library and complete the RNA sequencing. Data analyses will be performed using appropriate bioinformatic packages. We will integrate differential expression data with the molecular signature to assess the potential response of patients to IC. By means of this analysis, we will explore essential parts of the LHSCC molecular-genetic, immune regulation, and chemo-response picture.

Concurrently, baseline imaging will undergo radiomic analysis, as imaging quantitative features might capture distinct phenotypic differences of tumors, translating into prognostic/predictive features.

The combined analysis of all these data will allow a multi-scale profiling and predictive modeling of IC response and survival outcomes. This is foreseen to lead to the creation of a clinical decision support system (CDSS) able to personalize treatments for each patient.

The treatment decision will depend on integrating the radiogenomic signature with the patient’s clinical data in this multiomic signature.

These integrated data will identify potential complete responders or non-complete responders to standard chemotherapy. All patients enrolled in the study will undergo a first cycle of IC (platinum + taxane). In the meanwhile, genomic and radiomic analysis on the tumor biopsy will be carried out. If the signature suggests the patient is highly probably could achieve a complete response to chemotherapy, then he/she will receive the TPF chemotherapeutic regimen (1 cycle, taxane + platinum + 5-fluorouracil). However, if the signature indicates a lack of complete response to chemotherapy alone, it is conceivable that tumor escape via immune-related pathways may happen. In such cases, one cycle of immune checkpoint inhibitor will be combined with induction chemotherapy (platinum- and taxane-based). In case the signature predicts stable/progressive disease (and there is no response after the 1st cycle of platinum + taxane), the patient will be offered TL.

Patients in both IC treatment groups will undergo two cycles of induction treatment. After the second cycle, clinical (video laryngoscopy) and radiological (CT) restaging will occur. If there is no radiological/clinical response to the study treatment, the patient will be offered TL followed by postoperative radiation based on histological risk factors. In cases of partial response, full-dose curative IMRT with concurrent weekly cisplatin will be administered. For complete or near-complete responses, full-dose curative IMRT without concurrent chemotherapy will be given.

The main hypothesis behind this project is that patient selection remains crucial to improving outcomes, avoiding useless treatments, and reducing long-term toxicities.

The main objectives of the study are to evaluate laryngo-esophageal dysfunction-free survival (LEDFS), defined as being disease-free with functioning larynx in place and without tracheotomy or feeding tube, to evaluate the feasibility of a tailored systemic approach for a laryngeal preservation strategy, defined as % of patients enrolled that complete the study protocol treatment, to verify the 3 months the post-treatment proportion of laryngeal preservation, defined as the % of the patient not undergoing TL, to assess the radiological overall response rate (partial response, i.e. reduction of more than 50% of primary tumor volume + complete response) after induction treatment, to verify the OS of the whole cohort, to assess the safety of study treatment, to assess QoL using patient-reported outcomes measures (PROMs), by applying standardized validated instruments at each follow-up visit.

The main perspective for the future is to identify prognostic/predictive biomarkers based on translational analysis.

### 6.2 Cost-effectiveness analysis (CEA)

The PRESERVE prospective trial for tailored treatment may be the instrument to collect data on clinical outcomes and healthcare resource utilization (e.g., visits, drugs, etc.), while an *ad hoc* developed socio-economic questionnaire can be administered at each visit to patients to collect direct non-healthcare resource utilization and productivity losses. Health-related quality of life (HRQoL) data can be retrieved through validated questionnaires (e.g., EuroQoL) (58), directly administered to the patients at each visit. For non-tailored treatment, healthcare and non-healthcare resource consumption, productivity losses, clinical outcomes, and HRQoL data may be retrieved through the analysis of available databases, possibly integrated by a literature search. A cost-effectiveness model can be developed and populated for tailored treatment options, with data collected within the trial’s time horizon. Anyway, the model will be able to provide projections of costs and health outcomes over a lifetime landscape to capture any differences between the two options considered.

What can be foreseen from the CEA is that tailored treatment may be more costly but also more effective than the non-tailored option. In this case, the incremental cost-effectiveness ratio is calculated and then compared to a cost-effectiveness threshold to show the trade-off involved in choosing among the two treatment strategies. If the CEA provides evidence about the cost-effectiveness of tailored versus non-tailored IC treatment, this means that tailored IC within the LP approach can maximize benefits to society, patients, and their families and may open the way to the immediate diffusion of up-to-date medical research into the clinical practice.

## 7 Future perspectives

Different studies are investigating new drugs in LP, including ICIs, and a few of them have been presented. The addition of new drugs to standard induction treatment will hopefully improve the overall response rate, the LP rate and survival outcomes. However, it will still not help us understand the inner mechanisms of treatment resistance or provide prospective predictors of benefit from LP strategies in LA-LHSCC. As previously shown, to date, only limited clinical, pathological, and molecular predictors are available but come with rather low accuracy, may bring substantial risks for the patient or are not validated prospectively in independent cohorts. Other potential predictors, such as radiomics, seem promising, but the existing literature is very scant (52). To the best of our knowledge, a study aimed at unraveling a clinical and molecular signature of responders to IC has never been set up so far.

However, one should consider that overall survival is the main outcome of interest, and research on laryngeal preservation therapy should be conducted without compromising it. Therefore, the role of the multidisciplinary team having all the expertise in the treatment of laryngeal cancer is crucial in the selection of patient for organ preservation approaches.

## Author contributions

DM: Conceptualization, Methodology, Project administration, Writing – original draft, Writing – review & editing. GW: Writing – original draft, Writing – review & editing. DS: Writing – original draft, Writing – review & editing. AP: Writing – original draft, Writing – review & editing. MP: Writing – original draft, Writing – review & editing. RM: Writing – original draft, Writing – review & editing. MC: Writing – original draft, Writing – review & editing. AM: Writing – original draft, Writing – review & editing. SCh: Writing – original draft, Writing – review & editing. SCa: Writing – original draft, Writing – review & editing. YZ: Writing – original draft, Writing – review & editing. CR: Writing – original draft, Writing – review & editing. RT: Writing – original draft, Writing – review & editing. ES: Writing – original draft, Writing – review & editing. LL: Writing – original draft, Writing – review & editing. CG: Writing – original draft, Writing – review & editing. KK: Writing – original draft, Writing – review & editing. EH: Writing – original draft, Writing – review & editing. AD: Writing – original draft, Writing – review & editing. CP: Writing – original draft, Writing – review & editing. PB: Conceptualization, Methodology, Project administration, Writing – original draft, Writing – review & editing.
